# Extramedullary intracardiac multiple myeloma misdiagnosed as a thrombus: a case report

**DOI:** 10.1186/s12893-021-01377-y

**Published:** 2021-10-24

**Authors:** Ling Peng, Rurong Wang

**Affiliations:** Department of Anesthesiology, West China Hospital, Sichuan University, 37 Guo Xue Xiang, Chengdu, 610041 China

**Keywords:** Multiple myeloma, Extramedullary intracardiac plasmacytoma, Thrombus

## Abstract

**Background:**

Extramedullary intracardiac multiple myeloma (MM) is extremely rare. Patients with extramedullary intracardiac MM may suffer from a poor prognosis. Experience in the diagnosis and therapy of cardiac involvement in MM is limited. Herein, we describe a 67-year-old male with extramedullary intracardiac MM who was initially misdiagnosed with a thrombus.

**Case presentation:**

A 67-year-old male was admitted for exertional dyspnea and fatigue. The patient was diagnosed with MM one year earlier and had complete remission after chemotherapy. He was implanted with a permanent pacemaker two months prior due to sick sinus syndrome. After this admission, transthoracic echocardiography (TTE) and computed tomography (CT) confirmed the existence of a large right atrial mass extending to the superior and inferior vena cava. We initially considered the right atrial mass as a thrombus and performed surgical treatment for the patient. The surgical intervention partially relieved the obstruction of the superior and inferior vena cava and improved hemodynamics. Postoperative pathological examination of the right atrial mass suggested malignant plasmacytoma associated with MM. After recovery from the surgery, the patient received one cycle of chemotherapy. A follow-up of seven months revealed that our patient was still alive with a good general condition.

**Conclusions:**

Increasing the awareness of extramedullary intracardiac lesions in patients with MM is warranted. Our case confirmed that surgical intervention followed by adjuvant chemotherapy could improve the patient’s hemodynamics and achieve remission of cardiac symptoms.

## Background

Multiple myeloma (MM) is a malignant disease of plasma cells commonly associated with bone marrow plasmacytosis. The incidence of extramedullary lesions in MM is approximately 6–20% [1, 2]. However, cardiac involvement associated with MM is a rare manifestation with an incidence of less than 1% [1–3]. Thus, the experience in the diagnosis and management of cardiac involvement in MM is limited. Here, we present an MM patient whose large right atrial extramedullary plasmacytoma was initially misdiagnosed as a thrombus.

## Case presentation

### Clinical history

**Fig. 1:**
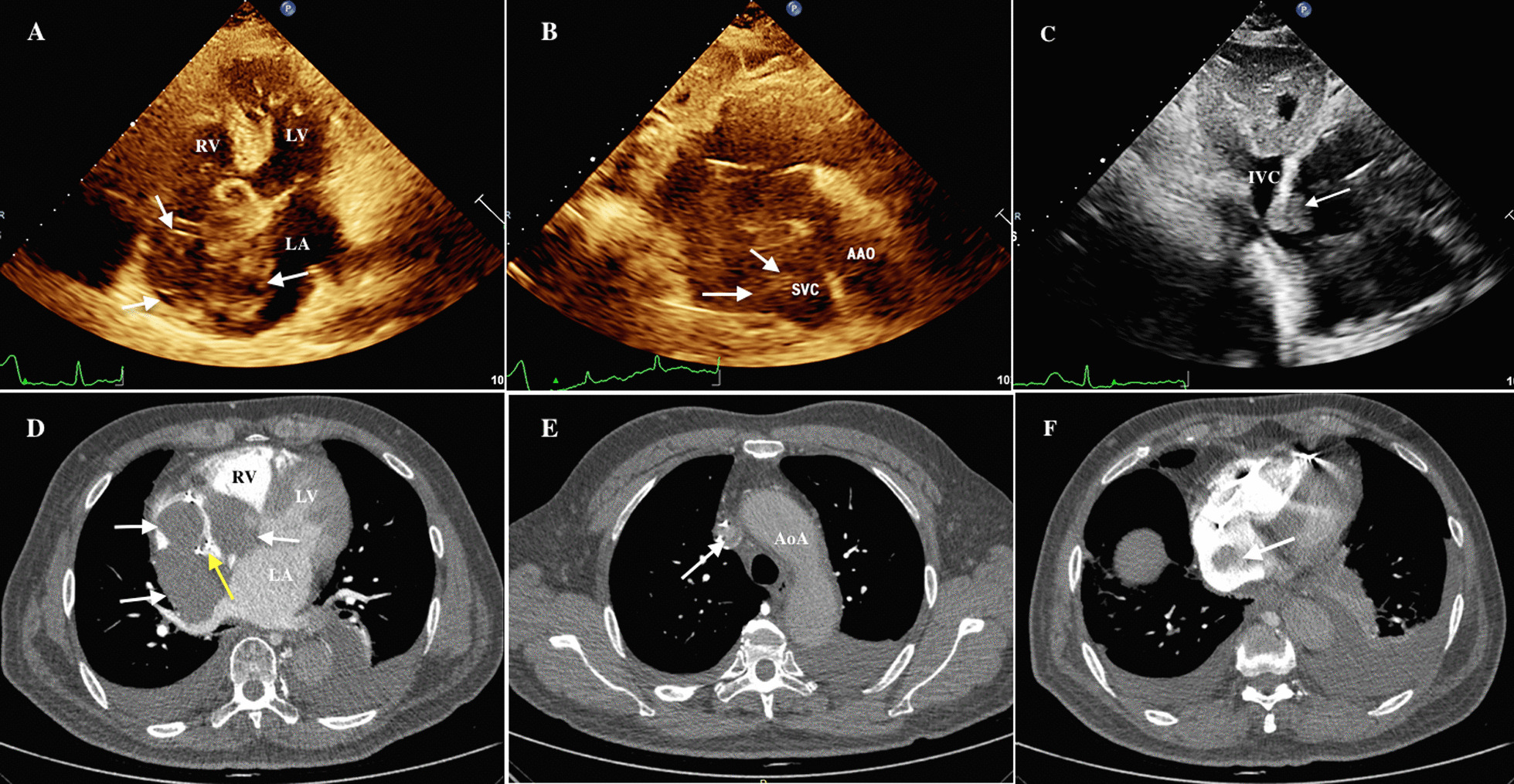
Echo view: **a** The arrows indicate that the large mass occupied most of the RA. **b** The arrows indicate that the mass extended into the IVC. **c** The arrow indicates that the mass extended into the SVC. Transverse view of CTA: **d**, **e** and **f** The arrows demonstrate the large mass in the RA extending into the IVC and SVC. The yellow arrow indicates the mass surrounding the pacemaker lead (**d**). RA, right atrium; RV, right ventricle; LA, left atrium; LV, left ventricle; AAO, ascending aorta; SVC, superior vena cava; IVC, inferior vena cava; AoA, aortic arch; CTA, computed tomographic angiography

A 67-year-old male was admitted for exertional dyspnea and fatigue. He was diagnosed with MM one year earlier. He had received chemotherapy with bortezomib, cyclophosphamide, and dexamethasone, achieving nearly complete remission without maintenance therapy. Two months earlier, he was diagnosed with sick sinus syndrome due to syncope and was implanted with a permanent pacemaker. After this admission, the patient had a body temperature of 36 °C, blood pressure of 119/72 mmHg, pulse rate of 74 beats per min, and respiratory rate of 20 beats per min. Physical examination revealed mild edema on his face and lower extremity. Slightly distended jugular veins were observed. The electrocardiogram revealed atrial fibrillation. Transthoracic echocardiography (TTE) showed a significant and irregularly shaped mass filling the right atrium (RA) (Fig. 1a) and obstructing the right ventricular inflow tract. The mass also extended into the superior vena cava (SVC) and inferior vena cava (IVC), resulting in almost complete occlusion of the SVC and partial obstruction of the IVC (Fig. 1b and c). A computed tomography (CT) scan also demonstrated a large mass in the RA extending to the SVC and IVC (Fig. 1d–f). The large mass surrounded the pacemaker lead (Fig. 1d). Bilateral pleural effusions were also observed. Angiography was negative for pulmonary embolism. The laboratory test showed a white blood cell count of 4.5 × 10^9^/L, hemoglobin level of 127 g/L, platelet count of 119 × 10^9^/L, creatinine level of 108 μmol/L, and alanine aminotransferase level of 57.7 U/L.

### Treatment

**Fig. 2:**
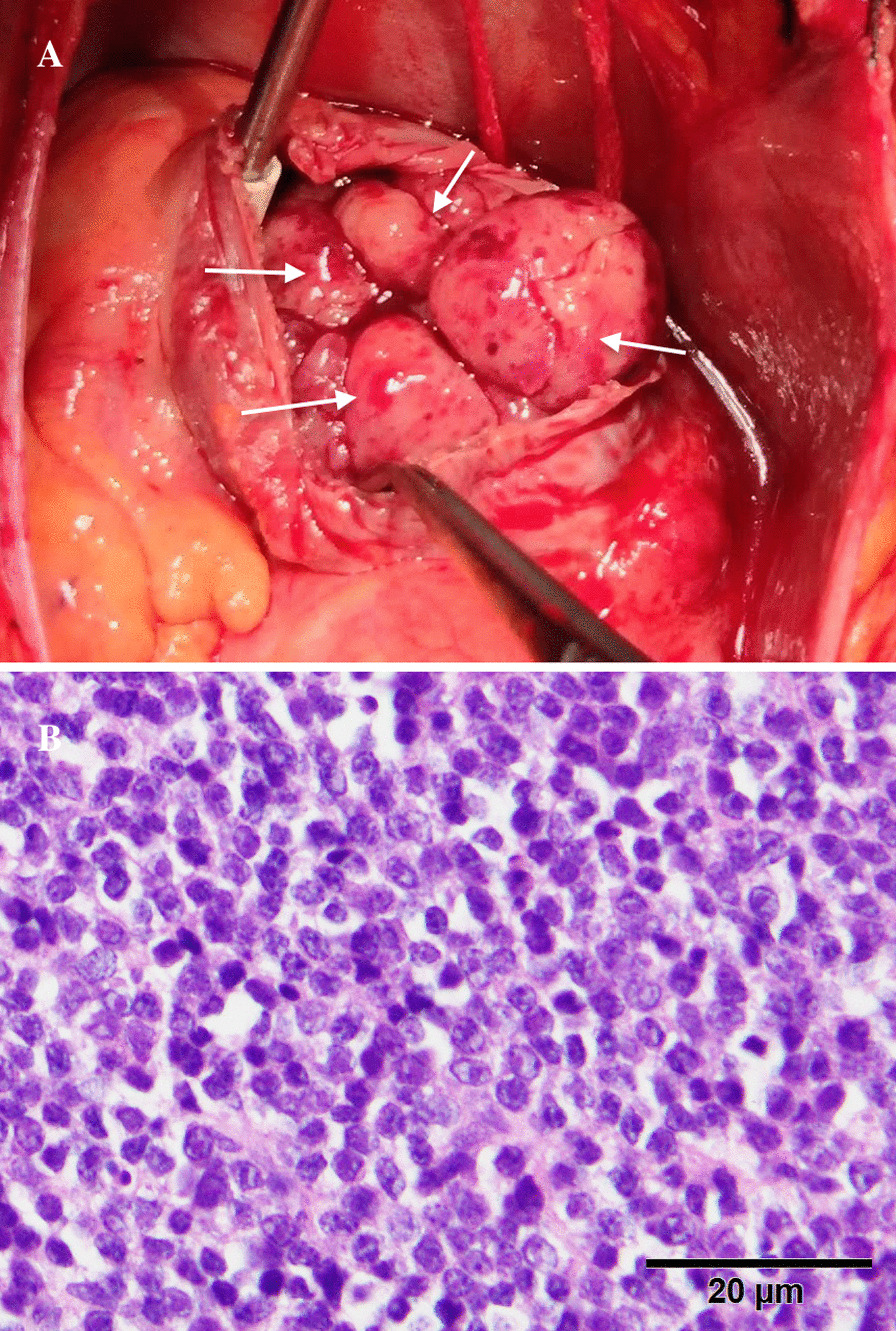
**a** Intraoperative findings: the mass occupied most of the right atrium. **b** Postoperative histologic specimens of the mass showed atypical immature plasma cells

Because the patient was in a stable course of MM and the pacemaker led through his SVC and RA, we initially considered the mass to be a right atrial thrombus. The patient underwent right atrial mass resection under cardiopulmonary bypass. Intraoperative findings revealed multiple lobulated masses occupying most of the RA (Fig. 2a), extending to the SVC and IVC. The mass also infiltrated the free wall of the RA, especially “freezing” the anterolateral wall of the RA. Intraoperative rapid pathological examination of the mass indicated a malignant tumor. The large mass in the RA could not be completely removed due to infiltration of the atrial wall. Therefore, a partial tumor and the anterolateral wall of the RA were resected to relieve the obstruction of the SVC, IVC, and right ventricular inflow tract. Then, the incision of the RA was reconstructed with a pericardial patch. The patient recovered uneventfully after the surgery. One month later, he received one cycle of chemotherapy with bortezomib, cyclophosphamide, and dexamethasone.

### Pathology and diagnosis

Postoperative pathological examination of the right atrial mass suggested malignant plasmacytoma associated with MM. Immunocytochemical staining of the tumor sections indicated atypical immature plasma cells positive for CD138, CD38, CyclinD1, and λ, and negative for CD20, CD79a, and CD10 (Fig. 2b).

### Outcome

After follow-up for seven months, our patient was still alive with a considerably good health condition after surgery. His echocardiography showed no intracardiac relapse of MM.

## Discussion and conclusion

Extramedullary cardiac plasmacytoma is extremely rare in patients with MM. Cardiac involvement of MM includes pericardial effusion [4], pericardial mass [5], intracardiac mass [2, 3, 6, 7], and myocardial infiltration [8, 9]. Extramedullary cardiac plasmacytoma may be either the initial or secondary presentation of MM [3]. A previous study suggested that extramedullary involvement of MM is relatively common in patients after receiving hematopoietic stem cell transplantation [4]. However, our patient did not receive hematopoietic stem cell transplantation during MM therapy.

The clinical symptoms of extramedullary intracardiac MM are nonspecific, depending on the localization and size of the mass. Patients might present with pericardial tamponade, arrhythmia, SVC syndrome, or severe heart failure [5, 10]. In this case, the large right atrial mass resulted in symptoms of SVC obstruction and right heart dysfunction in the patient. His previous sinus node dysfunction might also be associated with myocardial infiltration of the intracardiac plasmacytoma.

The diagnosis of extramedullary intracardiac MM requires pathological examination by transvenous biopsy or after surgical resection [7, 11]. Echocardiography and CT are the most commonly utilized imaging modalities to present the size and shape of the intracardiac mass but not to distinguish intracardiac plasmacytoma from other more common masses, such as thrombus, myxoma, or lymphoma. Contrast echocardiography may facilitate the classification of intracardiac tumors and thrombi [12]. Positron emission tomography (PET)/CT and magnetic resonance imaging (MRI) may be helpful for characterizing the involvement of intramedullary and extramedullary lesions associated with MM [1]. In our case, contrast echocardiography was not performed, and cardiac MRI was also not performed due to the in situ pacemaker. We initially misdiagnosed the right atrial mass as a thrombus because of the patient's history of implants in the right heart and his stable course of MM within one year after chemotherapy. This case indicated that extramedullary intracardiac lesions should not be overlooked in patients with MM, even during the stable course.

Extramedullary intracardiac lesions in MM patients carry a poor prognosis. More than half of the patients die within two days to 15 months after diagnosis [4, 11, 13]. The optimal therapy or management for intracardiac plasmacytoma is not yet well defined. Surgical resection, radiotherapy, and chemotherapy, alone or in combination, have been reported in a few cases to significantly decrease the size of intracardiac plasmacytoma and control cardiovascular symptoms but not to achieve long-term success [3, 6, 9, 13]. Admittedly, the appraisal and data of therapeutic strategy and outcome are limited due to the rarity of this disease. Surgical intervention is usually palliative because the intracardiac plasmacytoma usually infiltrates the myocardium and cannot be removed entirely. However, surgical intervention could immediately improve hemodynamics [10, 13]. Our patient differed from the previous patients who received surgical intervention for intracardiac plasmacytomas in one crucial aspect. In our case, except for most of the mass in the right atrial cavity, the infiltrated anterolateral wall of the RA was also resected and reconstructed with a pericardial patch. Our surgical strategy relieved the obstruction of the SVC and right ventricular inflow tract to the greatest extent. The surgical intervention followed by adjuvant chemotherapy resulted in a complete resolution of our patient’s symptoms of dyspnea and fatigue.

Extramedullary MM is distinctly uncommon in the heart, but it most often involves the upper respiratory tract, soft tissue, and gastrointestinal tract [9]. The clinical symptoms of extramedullary MM depend on the organs of involvement. Despite recognition of the poor prognosis of extramedullary MM, treatment and outcomes have not been fully studied. The data on chemotherapy, radiotherapy, surgical resection, and hematopoietic stem cell transplantation are limited [9]. Multimodal therapy may be associated with superior outcomes but needs adequately powered future studies.

In conclusion, increasing the awareness of extramedullary intracardiac lesions in patients with MM is warranted. Our practice confirmed that surgical intervention followed by adjuvant chemotherapy could achieve remission of cardiac symptoms.

## Data Availability

All data generated or analyzed during this study are included in this published article.

## Abbreviations

MM: Multiple myeloma
RA: Right atrium
TTE: Transthoracic echocardiography
SVC: Superior vena cava
IVC: Inferior vena cava
CT: Computed tomography
PET: Positron emission tomography
MRI: Magnetic resonance imaging
CTA: Computed tomographic angiography
